# Non-alcoholic fatty liver disease is associated with higher levels of *objectively* measured sedentary behaviour and lower levels of physical activity than matched healthy controls

**DOI:** 10.1136/flgastro-2014-100432

**Published:** 2014-06-30

**Authors:** Kate Hallsworth, Christian Thoma, Sarah Moore, Thomas Ploetz, Quentin M Anstee, Roy Taylor, Christopher P Day, Michael I Trenell

**Affiliations:** 1 Institute of Cellular Medicine, Newcastle University, Newcastle upon Tyne, UK; 2 The School of Computing Science, Newcastle University, Newcastle upon Tyne, UK

**Keywords:** FATTY LIVER, NONALCOHOLIC STEATOHEPATITIS, OBESITY

## Abstract

**Background and aims:**

Physical activity is a key determinant of metabolic control and is recommended for people with non-alcoholic fatty liver disease (NAFLD), usually alongside weight loss and dietary change. To date, no studies have reported the relationship between *objectively* measured sedentary behaviour and physical activity, liver fat and metabolic control in people with NAFLD, limiting the potential to target sedentary behaviour in clinical practice. This study determined the level of sedentary behaviour and physical activity in people with NAFLD, and investigated links between physical activity, liver fat and glucose control.

**Methods:**

Sedentary behaviour, physical activity and energy expenditure were assessed in 37 adults with NAFLD using a validated multisensor array over 7 days. Liver fat and glucose control were assessed, respectively, by ^1^H-MRS and fasting blood samples. Patterns of sedentary behaviour were assessed by power law analyses of the lengths of sedentary bouts fitted from raw sedentary data. An age and sex-matched healthy control group wore the activity monitor for the same time period.

**Results:**

People with NAFLD spent approximately half an hour extra a day being sedentary (1318±68 vs1289±60 mins/day; p<0.05) and walked 18% fewer steps (8483±2926 vs 10377±3529 steps/day; p<0.01). As a consequence, active energy expenditure was reduced by 40% (432±258 vs 732±345 kcal/day; p<0.01) and total energy expenditure was lower in NAFLD (2690±440 vs 2901±511 kcal/day; p<0.01). Power law analyses of the lengths of sedentary bouts demonstrated that patients with NAFLD also have a lower number of transitions from being sedentary to active compared with controls (13±0.03 vs15±0.03%; p<0.05).

**Conclusions:**

People with NAFLD spend more time sedentary and undertake less physical activity on a daily basis than healthy controls. High levels of sedentary behaviour and low levels of physical activity represent a therapeutic target that may prevent progression of metabolic conditions and weight gain in people with NAFLD and should be considered in clinical care.

## Introduction

Non-alcoholic fatty liver disease (NAFLD) represents a spectrum of progressive disease, widely considered the hepatic manifestation of the metabolic syndrome. NAFLD is strongly associated with obesity, insulin resistance (IR)/Type 2 diabetes melitus (T2DM), dyslipidaemia and cardiovascular disease.^1^ ^2^ The prevalence of obesity and IR have increased, and with this, NAFLD has rapidly become the most common cause of abnormal liver biochemistry in many developed countries.^3^ ^4^ Physical activity is a key determinant of metabolic control and is commonly recommended for people with NAFLD, usually alongside weight loss and dietary change.^5^ Even though physical activity and exercise are recommended as part of treatment for NAFLD, there have been no large-scale studies with adequate statistical power to guide healthcare practitioners in prescribing exercise programmes or for generating physical activity guidelines for the management of these patients.^6^ Evidence for the benefit of physical activity comes from prospective studies showing that individuals who maintain a physically active lifestyle are less likely to develop IR, impaired glucose tolerance, or T2DM.^7–10^

Cross-sectional studies suggest that people with NAFLD have lower levels of physical activity than those without^11–13^ and are more prone to fatigue.^14^ Increasing sedentary behaviour is becoming a growing problem in the general population,^15^ and low levels of physical activity are compounded by an increase in physical inactivity. Sedentary behaviour, including activities such as sitting, is reported to be higher in people predisposed to the metabolic syndrome, excessive adiposity and T2DM.^16–19^ Not only is the total duration of sedentary time important for metabolic risk, but also the breaks in sedentary time, independent of total sedentary time.^20^ Consequently, increases in sedentary time could play a potential role in the development of, or predisposition towards NAFLD, independent of physical activity/exercise, and needs to be considered when introducing lifestyle interventions. Targeting a reversal of sedentary behaviour may also provide an additional therapeutic avenue to complement physical activity and exercise guidelines.

Previously, activity levels in people with NAFLD have only been measured and described using physical activity questionnaires. Self-reported physical activity levels have been shown to be lower in people with NAFLD than their ‘healthy’ counterparts,^11–13^ and links have been made between low cardiorespiratory fitness and NAFLD severity.^1^ ^21^ ^22^ However, these subjective methods in determining physical activity are also subject to reporting error, linked to recall and social desirability bias, and are inaccurate in determining frequency, duration and intensity of physical activity.^23^ The use of a multisensor array to objectively measure physical activity provides data in terms of energy expenditure and step count, and also allows for an in-depth assessment of activity patterns including determination of sedentary behaviour.

To date, no studies have reported *objectively* measured physical activity and sedentary behaviour in people with NAFLD, limiting the potential to target sedentary behaviour in clinical practice. This study determined the level of objectively measured physical activity and sedentary time, and investigated links between physical activity, liver fat and glucose control.

## Subjects and methods

Thirty-seven adults with clinically defined NAFLD were recruited to the study from the subspecialty NAFLD clinic at the Freeman Hospital, Newcastle upon Tyne, UK. These were unrelated patients with NAFLD, derived from a patient population originally identified as having ultrasonographically detected bright liver and abnormal liver biochemical tests. Alternate diagnoses were excluded, including increased alcohol intake (males and females consuming greater than 21/14 units of alcohol per week (>30/20 g/day ethanol), respectively), chronic viral hepatitis (hepatitis B and hepatitis C), autoimmune liver diseases, hereditary haemochromatosis, α1-antitrypsin deficiency, Wilson's disease and drug-induced liver disease. NAFLD was defined as >5% liver fat content on ^1^H-MRS. Further exclusion criteria included: implanted ferrous metal; insulin sensitising treatment or dietary change (for people with T2DM, diet and metformin, were acceptable for inclusion if stable for 6 months). Patients had no physical restriction for exercise determined by an exercise test. The control group were individually matched to patients by age, sex and within 3 BMI from a control sample of over 1000 healthy people screened by questionnaire to be free of any metabolic disease.

The study protocol was approved by County Durham and Tees Valley 2 Research Ethics Committee. All participants provided written informed consent. Visits were undertaken at the Clinical Research Facility, Royal Victoria Infirmary, or the Magnetic Resonance Centre, both in Newcastle upon Tyne, UK.

### Liver fat measurement

Magnetic resonance studies were performed using a 3.0 Tesla Philips Achieva scanner (Philips Medical Systems, Best, The Netherlands). Liver fat was measured by localised ^1^H-MRS (PRESS, TR/TR=3000 ms/35 ms, 3×3×3 cmvoxel, SENSE torso Array). Blinded quantification of the spectra (water and CH_2_ resonances) was performed using the java-based magnetic resonance user interface (jMRUI V.3.0).^24^ ^25^ Following manual first and second-order phase correction, spectra were analysed using a non-linear least squares algorithm (AMARES).^26^ Liver fat was expressed as a percentage of liver volume, corrected for proton density of water and lipid.^27^

### Physical activity

Physical activity and energy expenditure were assessed objectively using a multisensor array (SenseWear Pro_3_, Bodymedia, Pennsylvania, USA) previously validated in healthy adults.^28^ Volunteers were asked to wear the armband on their right upper arm for 7 days. All subjects were instructed to remove the armband only for bathing/showering purposes or any water-based activity. A subject's multisensor array data were acceptable for analysis if overall wear-time was ≥80% of the total time that they had the monitor in situ.

The following matrices of physical activity were derived from the multisensor array as units per day: total energy expenditure (TEE); active energy expenditure (AEE); average metabolic equivalents (MET); duration of physical activity (>3.0 METs); duration of moderate physical activity (3.0–5.9 METs); duration of vigorous activity (6.0–9.0 METs); duration of very vigorous activity (≥9.0 METs); number of steps; and duration of monitor worn.

### Sedentary behaviour

Total sedentary duration was classed as total time spent in activities ≤2.9 METs, excluding sleep. Patterns of sedentary behaviour were assessed by power law analyses of the lengths of sedentary bouts fitted from raw sedentary data, as described in more detail previously.^29^ Briefly, the density p(x) of sedentary bouts in a time bin width d(x) was plotted against the bout length x on a logarithmic scale to derive power distribution (equation 1) from the shape of the histogram with respect to their length 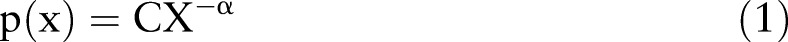


The type of sedentary distribution characterised by the exponent _α_ (equation 2), can quantify different sedentary behaviour strategies, with a lower _α_ indicating that subjects accumulate sedentary time with a larger proportion of long sedentary bouts: 
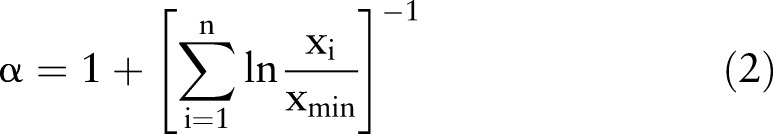


From these power distributions, Lorentz curves were calculated where the fraction W_x_ of the total sedentary time that is accumulated in bouts longer than any sedentary period of length x:
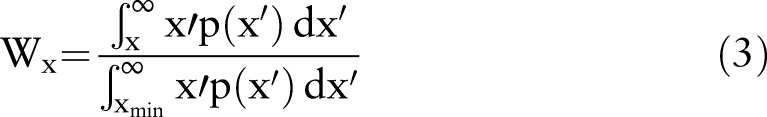


The curves are then plotted as Wx/p(x) pairs for each patient and control. Activity patterns were also assessed by assessing transitions from being inactive to active, and normalised by the length of the recording, termed ‘Sedentary to Active Transitions’. These data are presented as a percentage of the activity data per day.

Volunteers completed the validated^30^ International Physical Activity Questionnaire (IPAQ) to determine levels of physical activity and sit time after wearing the monitor for 7 days. The IPAQ includes four activity domains: job-related physical activity, transportation, housework (including house maintenance and caring for the family), recreation and leisure time activity. The IPAQ was scored using the guidelines produced by The IPAQ Group (http://www.ipaq.ki.se/scoring.pdf).

### Anthropometry

Bodyweight (kg) and height (cm) were measured using an electronic scale and stadiometer, respectively, (SECA, Birmingham, UK).

### Glucose control and liver enzymes

In the NAFLD group, a blood sample was taken from a forearm vein following an overnight fast (>8 h). Whole blood glucose was measured immediately (YSI 2300 Stat Plus-D, Yellow Springs Instruments, Yellow Springs, Ohio, USA). HbA1c was measured using a TOSOH HLC-723G7 (Tosoh Corporation, Tokyo, Japan) and ALT using a Roche Modular P and test kits (Roche Diagnostics, Burgess Hill, UK) in a Clinical Pathology Accredited laboratory (Newcastle Upon Tyne Hospital NHS Foundation Trust, Department of Clinical Biochemistry).

### Statistical analysis

Statistical analysis was performed using SPSS V.19 (SPSS, Chicago, USA). Between-group differences were evaluated using a paired t test, and Pearson's correlation was used to investigate associations between variables. Multivariate analyses were undertaken to control for BMI and age with respect to NAFLD. Statistical significance was set at a conservative threshold of p<0.01 to allow for multiple comparisons. Data are mean±SD.

## Results

The groups were well matched for age and sex (table 1). Weight and BMI were significantly higher in the NAFLD group when compared with controls (table 1). Liver fat content was 13±7%, ALT levels 55±33 UL^−1^, HbA1c 6.0±0.8% and fasting glucose 5.4±1.6 mmol/L in the NAFLD group. The control group did not self-report any disease.

**Table 1:** Patient and control demographics

	NAFLD	Control	p Value
Age (years)	53±13	52±12	0.20
Sex (M/F)	32/5	32/5	1.0
Weight (kg)	93±12	86±13	<0.05
BMI (kg m^2^)	32±4	28±4	<0.05
Liver fat (%)	13±7	N/A	
ALT (UL^−1^)	55±33	N/A	
HbA1c (%)	6.0±0.8	N/A	
Fasting plasma glucose (mmol/L)	5.4±1.6	N/A	

NAFLD, non-alcoholic fatty liver disease.

**Figure 1:**
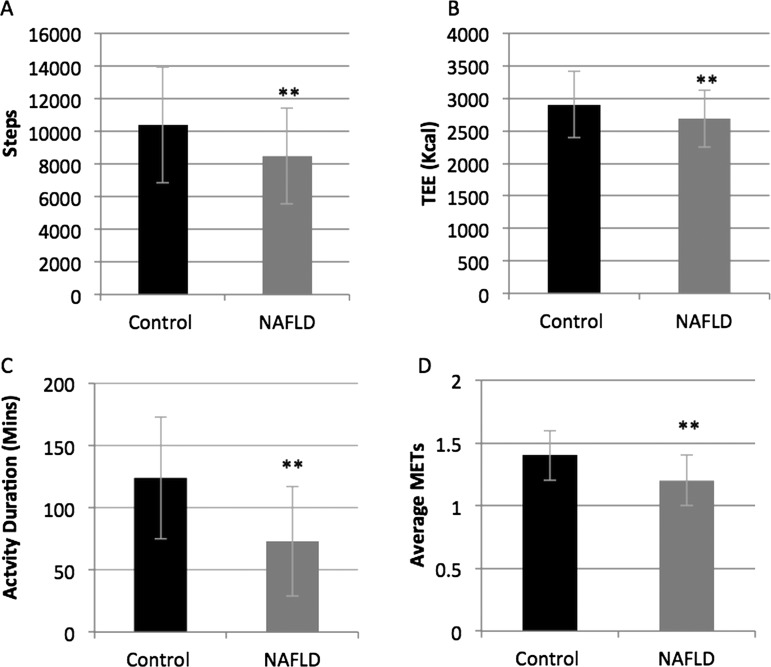
Objectively measured physical activity levels were lower in non-alcoholic fatty liver disease (NAFLD) compared with healthy controls (data reported as daily means (SD)). (A) Steps. (B) Total energy expenditure. (C) Physical activity duration. (D) Average MET levels.

**Figure 2:**
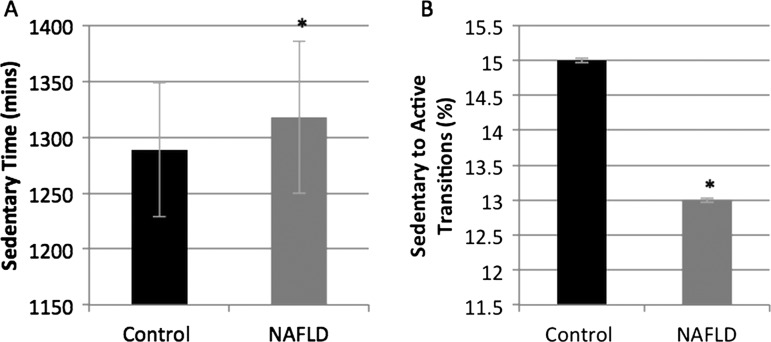
Sedentary time was higher in non-alcoholic fatty liver disease (NAFLD) than healthy controls with fewer sedentary to active transitions (data reported as daily means (SD)). (A) Sedentary time. (B) Sedentary to active transitions.

As summarised in table 2, the average number of steps taken each day was significantly fewer in NAFLD compared with controls (8483±2926 vs 10377±3529 steps/day; p<0.01; figure 1A) as was total daily energy expenditure (2690±440 vs 2901±511 kcal/day; p<0.01; figure 1B). Average daily MET levels were significantly lower in the NAFLD group when compared with controls (1.2±0.2 vs 1.4±0.2 METs; p<0.01; figure 1D), as was AEE (classed as activity of >3.0 METs: 432±258 vs 732±345 kcal; p<0.01). People with NAFLD spent less time performing physical activity of any intensity (73±44 vs 124±49 min/day; p<0.01; figure 1C) than the controls, and a significant difference was also observed between the groups when the physical activity was divided up into intensity levels (table 1). Sedentary time, classed as activities up to 3.0 METs, was not statistically significantly different between the groups, but was higher in the NAFLD group (1318±68 vs 1289±60 min/day; p=0.047; figure 2A).

**Table 2:** Physical activity data (data reported as daily means (SD))

Objective measures of physical activity (multisensor array)	NAFLD (n=37)	Control (n=37)	p Value
Duration on body (min) Percentage wear time	1390 (57) 96.5%	1409 (20) 97.8%	0.053
Lying (min)	476 (71)	482 (99)	0.787
TEE (kcal)	2690 (440)	2901 (511)	0.009
Steps	8483 (2926)	10377 (3529)	0.011
Average METs	1.2 (0.2)	1.4 (0.2)	0.001
Sedentary time (min)	1318 (68)	1289 (60)	0.047
AEE (kcal)	432 (258)	732 (345)	0.001
Physical activity duration (min)	73 (44)	124 (49)	0.001
Moderate activity (min)	71 (43)	109 (47)	0.001
Vigorous activity (min)	2 (4)	5 (7)	0.004
Very vigorous activity (min)	0 (2)	3 (9)	0.027
Subjective measures of physical activity (IPAQ)			
Mean daily MET-minutes	5806 (5635)	8783 (8968)	0.267
Mean daily sitting time (mins)	364 (182)	277 (107)	0.131

AEE, active energy expenditure; IPAQ, International Physical Activity Questionnaire; METs, metabolic equivalents, NAFLD, non-alcoholic fatty liver disease; TEE, total energy expenditure.

### Sedentary activity

Distribution analyses of the lengths of sedentary bouts demonstrate that patients with NAFLD have the same duration of sedentary bouts (Lorentz area under curve (AUC) 0.19±0.03 vs 0.18±0.02; p=0.106) as their healthy counterparts. The number of transitions from being sedentary to active were lower in patients with NAFLD compared with controls, but just failed to achieve statistical significance (13±0.03 vs 15±0.03%; p=0.021; figure 2B).

Using the self-reported IPAQ, people with NAFLD reported lower levels of physical activity and more time spent sitting than their healthy counterparts (see table 2). There was little correlation between the daily TEE recorded by the multisensor array and self-reported physical activity levels in the IPAQ across the whole group (r=−0.192; p=0.216). Sedentary time measured by the multisensor array was not associated with sitting time reported in the IPAQ (r=0.278; p=0.071).

Higher BMI was associated with lower average METs (r=−0.496; p<0.01), shorter physical activity duration (r=−0.494; p<0.01) and less moderate (r=−0.457; p<0.01) and vigorous activity undertaken (r=−0.445; p<0.01) in NAFLD. A trend towards a positive correlation between BMI and sedentary time was observed, however, this did not reach statistical significance (r=0.306; p=0.065). There was no correlation between liver fat, fasting glucose, HbA1c and ALT with any of the physical activity parameters measured by the multisensor array within the NAFLD group. Multivariate analyses were undertaken to control for BMI and age with respect to NAFLD. This showed that these factors contribute to lower activity levels and higher sedentary behaviour.

## Discussion

This is the first study to *objectively* measure sedentary behaviour and physical activity levels in adults with clinically defined NAFLD, and to use this data to investigate the relationship between physical activity, liver fat and metabolic control. The data reveals that people with NAFLD achieved lower levels of physical activity and spent more time sedentary than healthy controls. Levels of physical activity or sedentary behaviour were not associated with the severity of liver fat or glucose control in this small well-characterised group.

The present data highlights that people with NAFLD undertake less daily physical activity, by TEE, AEE and steps, than their healthy counterparts. Previous reports from self-report questionnaire reports also reveal that NAFLD is associated with lower levels of physical activity.^12^ ^13^ ^31^ However, questionnaires have significant limitations and are subject to recall and social desirability bias, and are inaccurate in determining frequency, duration and intensity of physical activity.^23^ The poor associations between objective and subjective reports of physical activity in the present study highlight the importance of objectively assessing physical activity. The link between physical activity and liver fat in previous research highlights the positive effects of a physically active lifestyle upon IR, impaired glucose tolerance and T2DM.^32–34^ Physical activity should, theoretically, aid the prevention and/or progression of NAFLD through its reciprocal relationship with glucose control, and has been shown to improve liver histology when used as part of a lifestyle intervention in conjunction with diet and weight loss.^35^

People with NAFLD also undertake less moderate and vigorous activity than healthy controls. The lower levels of these higher-intensity activities may have implications, as the intensity of the activity may also play a key role in improving metabolic control. However, the reports demonstrating that higher intensity activities/exercises are linked to improvements in metabolic control are not unequivocal. One meta-analysis found exercise intensity was not associated with a difference in HbA1c in people with T2DM.^10^ However, when using resistance training independently, moderate high-intensities were associated with greater improvements in muscle bulk and overall glucose control,^36^ and high-intensity interval training was shown to improve hyperglycaemia in patients with T2DM.^37^ Harrison and Day^5^ speculate that moderate exercise, performed 3–4 times per week, expending about 400 kcal each time appears adequate to augment improvement in the metabolic profiles of patients with NAFLD. However, although useful, the evidence underlying these clinical guidelines is lacking. There is no clear evidence on which exercise approach is best in improving metabolic control with recent data also suggesting benefit from resistance rather than cardiovascular exercise.^34^

A novel observation in the present dataset is that adults with NAFLD spend more time being sedentary than those without fatty liver. Sedentary behaviour or physical inactivity is a growing health problem, silently putting people at heightened risk from a host of chronic diseases.^15^ ^38^ This increase in physical inactivity may compound the detrimental health effects caused by lack of physical activity. In the present study, adults with NAFLD accumulated 22 h per day of sedentary activity. Sedentary behaviours involving sitting or lying down are characterised by a low MET value of less than 3, and are related adversely to metabolic biomarkers and to poorer health outcomes.^39^ Adults with NAFLD also had fewer sedentary to active transitions (ie, breaks in sedentary time) than the healthy controls. Sitting for prolonged periods reduces the opportunity for cumulative energy expenditure produced by muscle contractions as we move around throughout the day,^40^ and impairs the exercise/muscle contraction-stimulated uptake of glucose from the circulation and lipoprotein lipase activity thus hampering fat handling. Even if adults meet the public health guideline for leisure-time physical activity, they may have a high risk of becoming overweight or developing metabolic disorders if they spend a large amount of time in sedentary behaviours during the rest of the day.^18^ ^39^ Combined, these results demonstrate for the first time that sedentary behaviour is prominent in NAFLD—targeting these periods of inactivity may constitute an effective means of improving liver fat.

In patients with NAFLD, BMI was negatively correlated with objectively measured markers of increasing physical activity and positively associated with sedentary time. In obesity, studies have shown similar findings^41–43^ whereby the more overweight/obese people are, the less physical activity they undertake, which drives the vicious cycle of increasing weight gain. Given the strong link between BMI and NAFLD, it is possible that the main effect of physical activity on NAFLD is through its relationship with body weight. This observation also highlights the potential for reducing sedentary behaviour and increasing physical activity in the prevention of weight gain, a major driver for the development of NAFLD and poor metabolic control. It should be noted, however, that the relative small sample size may not be sufficient to see other relationships.

The data produced by the multisensor array provides useful insights into free-living daily activity patterns in people with NAFLD. The MET levels provided also act as a guide as to the intensity of activity undertaken which allows clinicians to tailor advice to this. Volunteers found the monitors easy to use and unobtrusive, with little impact on daily activity. Adherence to wearing the monitor was high, as demonstrated by a mean percentage wear time of >96%. Limitations of these monitors are that they are not waterproof and thus need to be removed for any water-based activity. It should also be noted that the present study may be limited by the relatively small cohort sizes, the cross-sectional design which removes the ability to assign causality, and the absence of liver fat and blood sample measurements for control subjects.

### Clinical implications

The use of objective measures of physical activity and sedentary behaviour in the clinical environment may provide clinicians with a way to engage patients in discussion about activity/exercise. Data recorded can be used as a baseline measure from which to tailor subsequent physical activity counselling and build appropriate exercise programmes. Their use offers the opportunity to provide immediate feedback to patients when they return to clinic, by providing a short report or a more in-depth daily analysis of activity, from which discussions about lifestyle change and weight loss can materialise. Since the visual data being presented by the clinician represents the patient's actual day-to-day life, this may act as a valuable tool to aid in improving adherence, patient motivation and clinical outcomes.

In conclusion, people with NAFLD spent more time sedentary and less time physically active on a daily basis than people without fatty liver. Given the established relationship between sedentary behaviour and physical activity with metabolic regulation, weight gain and cardiovascular disease risk, high levels of sedentary behaviour and low levels of physical activity may represent effective therapeutic targets in the management of NAFLD. Often, patients are not aware how much physical activity or sedentary behaviour they actually engage in, so an objective measure will provide this feedback, and thus allow personal activity goals to be established in order to achieve their individual health targets. Combined, these data suggest that clinical care teams should consider the use of objective monitoring and targeting of sedentary behaviour and low levels of physical activity as a means to improve metabolism, prevent weight gain and delay disease progression in people with NAFLD.

**Significance of this study**

**What is already known in this topic?**

- A physically active lifestyle is important for good metabolic control and wellbeing.
- Self reported physical activity is reported to be lower in people with NAFLD than people without NAFLD.
- Sedentary behaviour (as opposed to physical activity) has also been shown to be a strong predictor of worsening metabolic control and cardiovascular disease but has not been measured in NAFLD.

**What this study adds?**

- This is the first study to objectively show that people with NAFLD are more sedentary than people without NAFLD.
- Objectively measured physical activity is also lower in people with NAFLD.

**How might it impact on clinical practice in the foreseeable future?**

- Sedentary behaviour may be an easier therapeutic target for clinical care teams to help patients target over more substantive behaviour changes.
